# Patterns of Cerebellar Gray Matter Atrophy Across Alzheimer’s Disease Progression

**DOI:** 10.3389/fncel.2018.00430

**Published:** 2018-11-20

**Authors:** Sofia Toniolo, Laura Serra, Giusy Olivito, Camillo Marra, Marco Bozzali, Mara Cercignani

**Affiliations:** ^1^Neuroimaging Laboratory, IRCCS Santa Lucia Foundation, Rome, Italy; ^2^Clinical Imaging Sciences Centre, Brighton and Sussex Medical School, Brighton, United Kingdom; ^3^Ataxia Laboratory, IRCCS Santa Lucia Foundation, Rome, Italy; ^4^Department of Psychology, Sapienza University of Rome, Rome, Italy; ^5^Institute of Neurology, Catholic University, Rome, Italy

**Keywords:** Alzheimer’s disease, mild cognitive impairment, voxel-based morphometry, SUIT, cerebellum, vermis, lobule VI, constructional apraxia

## Abstract

The role of the cerebellum in cognitive function has been broadly investigated in the last decades from an anatomical, clinical, and functional point of view and new evidence points toward a significant contribution of the posterior lobes of the cerebellum in cognition in Alzheimer’s disease (AD). In the present work we used SUIT-VBM (spatially unbiased infratentorial template, voxel-based morphometry) to perform an analysis of the pattern of cerebellar gray matter (GM) atrophy in amnestic mild cognitive impairment (a-MCI) and AD dementia patients compared to healthy subjects (HS), in order to follow the changes of non-motor features of cerebellar degeneration throughout disease progression. This template has been validated to guarantee a significant improvement in voxel-to-voxel alignment of the individual fissures and the deep cerebellar nuclei compared to Montreal Neurological Institute (MNI) whole-brain template. Our analysis shows a progression of cerebellar GM volume changes throughout a continuous spectrum from early to late clinical stages of AD. In particular vermis and paravermian areas of the anterior (I-V) and posterior (VI) lobes are involved since the a-MCI stage, with a later involvement of the hemispheric part of the posterior lobes (VI lobule) and Crus I in AD dementia patients only. These findings support the role of the cerebellum in higher-level functions, and whilst confirming previous data on the involvement of Crus I in AD dementia, provide new evidence of an involvement of the vermis in the early stages of the disease.

## Introduction

Alzheimer’s disease (AD) is the most prevalent cause of dementia worldwide, leading to a progressive loss of cognitive and functional abilities. The diagnostic criteria for AD have been recently revised by the International Working Group for New Research Criteria for the Diagnosis of AD to assimilate the technological and biochemical advances that enable us nowadays to diagnose AD with a high level of accuracy, even at the onset of the earliest clinical manifestations (Dubois et al., 2014). The loss of GM volume along with the progression from MCI to AD dementia has been widely investigated, showing an early impairment in parahippocampal gyrus, precuneus, posterior cingulate, frontal lobe, insula, and the cerebellum 12 months before AD dementia diagnosis (Spulber et al., 2012; Serra et al., 2016). Despite much attention has been recently given to the role of the cerebellum in AD (Jacobs et al., 2017), only few studies specifically tackled the pattern of the cerebellar GM atrophy and its contribution to cognitive decline alongside its clinical progression.

The role of the cerebellum in cognitive function has been broadly investigated in the last decades from an anatomical, clinical and functional point of view (Leggio and Olivito, 2018) since the original description of the CCAS by Schmahmann and Sherman (1998). This syndrome was originally described as a subset of symptoms due to the selective damage to the vermis and posterior lobe of the cerebellum. In those patients a selective impairment of executive functions such as planning, set-shifting, verbal fluency including agrammatism and dysprosodia, abstract reasoning and working memory, difficulties with spatial cognition including visual-spatial organization and memory and even personality change with blunting of affect or disinhibited and inappropriate behavior (up to the so called cerebellar mutism syndrome) were described (Schmahmann and Sherman, 1998). Moreover, deficits in executive functions, attentional processes, working memory and divided attention have been described after microsurgical treatment of tumors or hematomas in the cerebellum (Gottwald et al., 2004; De Ribaupierre et al., 2008; Grossauer et al., 2015).

The neuroanatomical basis of the cerebellar role in cognitive function was unraveled by the pioneer work by Kelly and Strick (2003) with rabies viral neurotracers, which discovered beside the motor cortico-cerebellar loop which connects the primary motor cortex (M1) to Purkinje cells in lobules IV–VI of the cerebellar cortex, a second closed loop involved in non-motor tasks which connects Brodmann area 46 (prefrontal cortex) to granule cells mainly in Crus II. Later on, a second non-motor circuit that links the inferior parietal cortex to the dentate nucleus of the cerebellum has been described (Clower et al., 2001, 2005; O’Reilly et al., 2010; Prevosto et al., 2010). This neuroanatomical finding is reflected by a functional point of view in a clear separation of the cerebellum in two distinct functional zones, a primary sensory-motor zone (Lobules V, VI, and VIII), which shows connections to motor, somatosensory, visual, and auditory cortices and a “supramodal” zone [Lobules VIIa (Crus I, and II)], which shows high connectivity with prefrontal and posterior-parietal cortex (Prevosto et al., 2010). Anyway, whether these functionally distinct features are reflected in a clinically detectable cognitive decline associated to a selective GM atrophy in the cerebellum has been still poorly investigated.

The first study which specifically tackled the topic of the GM atrophy pattern in the cerebellum of MCI and AD dementia patients by Thomann et al. (2008) showed smaller volumes in the right and left superior posterior lobe and right inferior posterior cerebellar lobes of AD dementia patients in comparison with MCI patients and healthy subject. A technical limitation of this study was that the cerebellum was parceled manually, which accounts for a significant lower accuracy and reproducibility due to a higher operator inter-variability (Magnotta et al., 2002). An automated and unbiased assessment such as VBM would be more attractive (Ashburner and Friston, 2000). However, the MNI space ICBM-152 (International Consortium for Brain Mapping) whole-brain template, typically used for VBM, provides limited contrast in the cerebellum and has been shown to be limited in accuracy in alignment of the cerebellar anatomical landmarks to standard space. In order to address the need for a more detailed analysis of the cerebellum, SUIT toolbox was developed with the intent to preserve much more of the cerebellar anatomical details (Diedrichsen et al., 2009). This template has been validated to guarantee a significant improvement in voxel-to-voxel alignment of the individual fissures and the deep cerebellar nuclei compared to MNI whole-brain template. Here we used SUIT-VBM to perform a detailed analysis of the pattern of cerebellar GM atrophy in patients with amnestic mild cognitive impairment (a-MCI) and AD dementia patients compared to HS, in order to follow the changes of non-motor aspects of cerebellar degeneration throughout disease progression. We decided to enroll only MCI patients with a prominent amnestic presentation (a-MCI, single and multiple domain) in order to increase the probability of including in the study patients that will evolve clinically in AD dementia (Chan et al., 2011).

## Materials and Methods

### Participants

One hundred forty nine participants were recruited from the Dementia unit of the Catholic University, Rome: 53 patients with probable AD dementia according to the new McKhann criteria of 2011 (McKhann et al., 2011), 62 amnestic MCI (a-MCI) patients (single and multiple domain) according to Albert Criteria of 2011 (Albert et al., 2011) and Petersen Criteria of 2014 (Petersen et al., 2014), and 34 HS. None of the HS showed evidence of cognitive deficits on neuropsychological testing. All participants underwent a complete clinical investigation, including medical history, neurological examination, a complete blood screening (including routine exams, thyroid hormones, level of B_12_).

Exclusion criteria for all subjects included contra-indication for magnetic resonance imaging (MRI), previous history of alcohol or substance abuse, focal brain lesions on brain imaging, significant neurological or psychiatric history and presence of major systemic illnesses. The subjects were equally gender, education, and age matched. Subjects were excluded if they had two or more hyperintense lesions >10 mm, or more than eight hyperintense lesions between 5 and 9 mm to exclude prominent vascular damage. The study was approved by the Ethical Committee of Santa Lucia Foundation and written informed consent was obtained from all participants before study initiation. All procedures performed in this study were in accordance with the 1964 Helsinki declaration and its later amendments or comparable ethical standards.

### Neuropsychological Assessment

All patients underwent a complete Neuropsychological battery before MRI. The mini-mental state examination (MMSE) was performed for assessing general cognitive status (Folstein et al., 1975; Magni et al., 1996), Rey 15-Words List Immediate recall (cut-off ≥ 28.5) and Delayed recall (cut-off ≥ 4.6) (Carlesimo et al., 1996) for verbal episodic long-term memory, Copy of drawings (cut-off ≥ 7.1) (Carlesimo et al., 1996) and Copy of drawings with landmarks (cut-off ≥ 61.8) (Carlesimo et al., 1996) for praxis, Digit span forward (cut-off ≥ 3.7) and back (Orsini et al., 1987) for verbal short term memory and Corsi blocking task forward (cut-off ≥ 3.5) and back (Orsini et al., 1987) for visuo-spatial short term memory, Phonological Word Fluency (cut-off ≥ 17.3) (Carlesimo et al., 1996) for executive functions, Naming of objects (cut-off ≥ 22) (Miceli et al., 1991) for language skills, Raven’s Colored Progressive Matrices (cut-off ≥ 18.9) (Carlesimo et al., 1996) for reasoning. Each test was adjusted for gender, age, and education for the neuropsychological statistical analyses.

### MRI Data Acquisition

Magnetic resonance imaging was done at 3T (Magnetom Allegra, Siemens, Erlangen, Germany) with the following sequences: dual echo turbo spin echo (TSE) (TSE TR = 6190 ms, TE = 12/109 ms), fast fluid attenuated inversion recovery (FLAIR) (TR = 8170 ms, TE = 96 ms), 3D modified driven equilibrium fierier Transform (MDEFT) scan, (TR = 1338 ms, TE 2,4 ms, Matrix = 256 × 224 × 176, in-plane FOV = 250 mm × 250 mm, slice thickness 1 mm). According to the inclusion criteria, TSE and FLAIR scans were reviewed to exclude the presence of remarkable macroscopic brain abnormalities. The cerebellum was pre-processed individually in SPM-8^1^, using SUIT, a dedicated toolbox that allows to extract and normalize the gray (GM) and white matter (WM) from the cerebellum. SUIT is a high-resolution atlas template of the human cerebellum and brainstem, spatially unbiased, which through automated non-linear normalization methods, provides a more accurate intersubject-alignment than current whole-brain methods (Diedrichsen et al., 2009). The data were segmented fully into GM and WM, prior to normalization to a cerebellar focused SUIT template. This may allow more accurate co-registration, and hence may permit a better GM-specific analysis of volume loss. Images were smoothed using an 8-mm FWHM Gaussian kernel.

### Statistical Analyses

Statistical analyses were performed on smoothed GM maps within the framework of the general linear model. A one-way ANOVA model was used for assessing between group differences in regional GM cerebellar volumes. Age, sex, and years of education were included as covariates in our analysis. In addition we also evaluated the association between GM volume and neuropsychological scores in a voxel-wise fashion. Regional differences and correlations were considered significant only if they survived after correction for multiple comparisons (Family wise error, FWE correction at cluster-level, *p* < 0.05 – clusters formed at uncorrected level with *p* < 0.001).

## Results

As shown in Table 1 there were no significant differences in demographic features among the three groups. As expected, AD dementia patients showed a statistically lower MMSE compared to a-MCI and HS patients, as well as lower scores in Rey 15 list immediate and delayed recall, Phonological verbal fluency, Digit span forward and back, Corsi blocking task forward, Copy of drawings and Copy of drawings with landmarks and Raven’s progressive matrices. The same pattern was observed in a-MCI patients with respect to HS, with the exception of Copy of drawings and Copy of drawings with landmarks, which did not reach statistically significance.

**Table 1:** Neuropsychological assessment.

	AD dementia	a-MCI	HS
	*N* = 53	*N* = 62	*N* = 34
Mean age (years)	75.3 (5.7)	70.2 (9.7)	69.2 (6.8)
Gender (F/M)	35/18	31/31	17/17
Mean years of formal education	11.3 (4.4)	11.6 (4.4)	12.8 (3.6)
MMSE	20.1 (3.3)*^§^	26.5 (2.2)^∧^	28.9 (1.6)
Rey 15 word list (immediate recall)	22.3 (4.5)*^§^	29.1 (5.7)^∧^	46.4 (8.6)
Rey 15 word list (delayed recall)	2.3 (2.1)*^§^	3.5 (2.5)^∧^	9.6 (8.3)
Phonological verbal fluency	18 (14.7)*^§^	30.9 (4)^∧^	37 (9)
Digit span forward	4.6 (1.2)*^§^	5.2 (5)^∧^	5.8 (1)
Digit span back	2.9 (0.6)*^§^	3.4 (1.4)^∧^	4.4 (0.7)
Corsi blocking task forward	3.6 (1.4)*^§^	4.4 (0.6)^∧^	5 (0.8)
Corsi blocking task back	2.8 (1.6)	3.6 (1)	4.6 (0.7)
Copy of drawings	6 (3.3)*^§^	9.9 (1.7)	10.9 (1.2)
Copy of drawings with landmarks	52.8 (20.5)*^§^	65.1 (13.6)	69.4 (0.7)
Naming	24.7 (5.9)	28.4 (1.9)	29.4 (0.7)
Raven’s progressive matrices	29.2 (8)*^§^	27.1 (4.9)^∧^	31.4 (3.7)


*^∗^AD dementia < HS, p-level < 0.05; ^∧^a-MCI < HS, p-level < 0.05; ^§^AD dementia < a-MCI, p-level < 0.05.*

### VBM Cerebellar Regions of GM Atrophy

**FIGURE 1:**
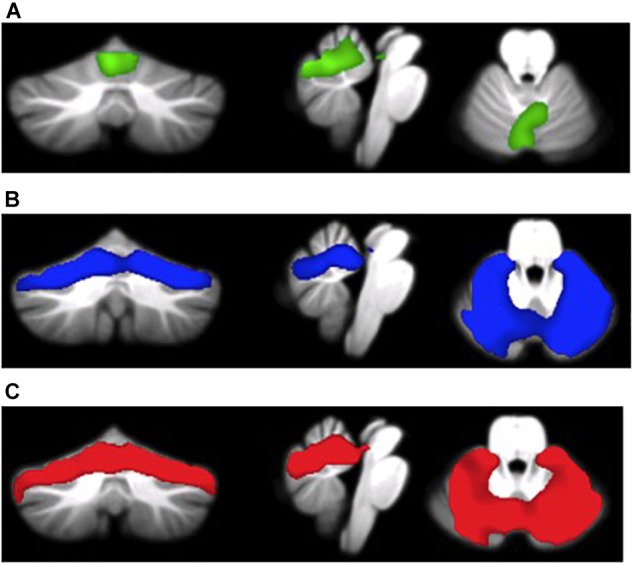
**(A)** MCI patients < HS (green overlay), **(B)** AD patients < MCI patients (blue overlay), and **(C)** AD patients < HS (red overlay).

As shown in Table 2 and Figures 1A–C, we found significant between-groups differences in cerebellar GM volumes. Specifically, a-MCI patients compared to HS showed a significant GM loss in vermis and paravermian lobules I–VI bilaterally (Figure 1A). AD dementia patients showed a significant GM loss in vermis and paravermian lobules I–VI, and also in lobules VI bilaterally and in Crus I compared to a-MCI (Figure 1B) and HS (Figure 1C).

**Table 2:** VBM atrophy MNI Coordinates and Brain regions.

	Cerebellar brain regions	Side	Cluster size	Coordinates			*Z*-score
					
				x	y	z	
a-MCI < HS	Vermis and paravermian Lobules I–VI	L, R	5734	-1	-74	-16	2.75
				5	-58	-17	2.70
				13	-40	-14	2.59
AD dementia < a-MCI	Vermis and paravermian lobules I–VI	L, R	49372	22	-32	-22	7.21
	VI lobules	L, R		24	-59	-17	7.14
	Crus I	L, R		30	-44	-22	7.11
AD dementia < HS	Vermis and paravermian lobules I–VI	L, R	52983	19	-35	-19	6.55
	VI lobules	L, R		39	-63	-23	6.49
	Crus I	L, R		-4	-51	-16	6.37


*Side: Left (L), Right (R). Cluster Size is displayed by voxel numbers. Coordinates refer to the MNI space coordinates. Z-scores were calculated by SPM, which is an automated mean of analyzing the p-values from the t statistics.*

### Correlations Between Neuropsychological Scores and Cerebellar Regions of GM Atrophy

**FIGURE 2:**
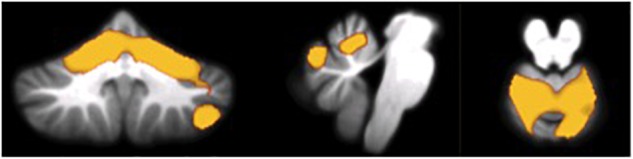
Voxel-wise correlations between scores at the Copy of drawings test and GM atrophy in AD patients.

**FIGURE 3:**
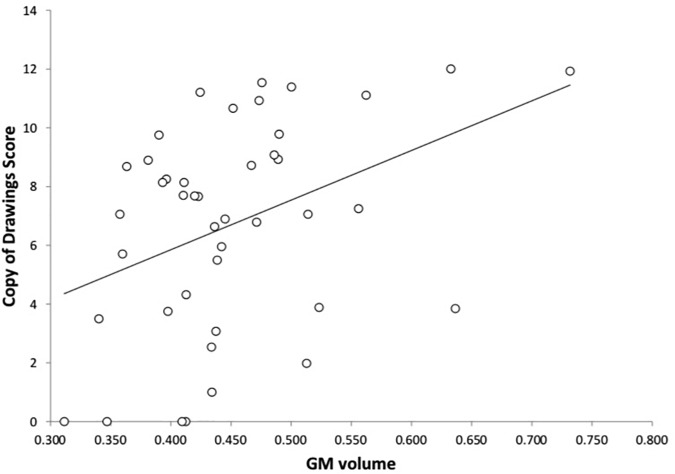
Correlation between the Copy of drawings test and GM volume in AD dementia patients. On the *y*-axis the scores at the Copy of drawings test, on the *x*-axis the mean GM voxel volumes expressed in mm^3^.

As shown in Figures 2, 3 and Table 3, in AD dementia patients we found a positive correlation between Copy of drawings test scores and GM loss in vermis and paravermian lobules I–VI, in lobules VI and VIIa (Crus I and II), and lobules VIIb bilaterally, *p*_unc_ = 0.031. No other significant correlations were found among the other tests and in the others diagnostic groups.

**Table 3:** Copy of drawings MNI coordinates and brain regions.

	Cerebellar brain region	Side	Cluster size	Coordinates			*Z*-score
					
				*x*	*y*	*z*	
Copy of drawings	Vermis and paravermian lobules I–VI	L, R	46518	13	-78	-19	3.41
	VIIa lobules (Crus I, Crus II)	L, R		-17	-74	-44	3.06
	VIIb lobules	L, R		-28	-74	-45	2.92


*Side: Left (L), Right (R). Cluster Size is displayed by voxel numbers. Coordinates refer to the MNI space coordinates. Z-scores were calculated by SPM, which is an automated mean of analyzing the p-values from the t statistics.*

## Discussion

The loss of GM volume along with the progression from MCI to AD has been previously stated, showing an early impairment in medial temporal lobe structures (hippocampus and parahippocampal areas) and precuneus as well as in cerebellar structures in MCI patients (Spulber et al., 2012) with a high prognostic value in MCI patients that will develop AD dementia (Serra et al., 2016). Nevertheless, studies focusing specifically on the pattern of GM loss in the cerebellum of AD population are scarce.

This study indicates a progression of cerebellar GM volume changes throughout a continuous spectrum from early to late clinical stages of AD. In particular vermis and paravermian areas of the anterior (I–V) and posterior (VI) lobes are involved since the a-MCI stage, with a later involvement of hemispheric posterior lobes (VI lobules and Crus I). Subsequently we would like to analyze the meaning of these findings according to their topographical localization.

What does vermian atrophy means in this context? The vermis is particularly vulnerable in the aging population (Luft, 1999), since older adults show a higher degree of GM loss in the vermis, with a rate of GM loss of approximately 4.59% per decade, nearly double than over the cerebellum as a whole (Yu et al., 2017). This finding is supported by anatomopathological data by Andersen et al that showed a selective reduction in the anterior lobe of the cerebellum corresponding to lobules I–V with age, with a 40% loss of Purkinje and granule cells and a 30% loss of global cortical volume selectively in the anterior lobe (Andersen et al., 2003). Continuing on the topic of age-related vermian cerebellar changes, Bernard at al raised the question whether, from a functional point of view, cortico-cerebellar connectivity would change with respect to age, since most fMRI studies tackling cerebellar functional activity are based on data collected from young subjects. According to their study, there is a disruption of cortico-cerebellar connectivity through the lifespan with a widespread decrease in the strength of the resting state networks in older adults, especially between the cerebellum and the striatum and between the cerebellar right hemisphere and vermis and the hippocampus and parahippocampal gyrus (Bernard et al., 2013). Nevertheless our study supports the idea that GM atrophy in the vermis is not merely age dependent, as our population sample showed no statistical differences in age between HS, a-MCI, and AD dementia patients. We also included age as a covariate in our analysis for avoiding any confounding age factors.

Gray Matter atrophy of the VI lobule in AD dementia patients is supported by previous VBM data (Colloby et al., 2014), but to our knowledge this is the first study to assess that the GM loss is present even in the early phase of the disease (a-MCI). From a functional point of view, there is evidence that the VI lobule has a clear involvement in non-motor functions of the cerebellum (Stoodley et al., 2010) with a selective activation of the right VI lobule through verb generation (Stoodley et al., 2012), left VI lobule during mental rotation tasks (Iglói et al., 2015), and bilateral activation through working memory tasks (Stoodley et al., 2012). The VI lobule could also be involved in the emotional processing of stimuli, since viewing arousing images activates lobules VI bilaterally (Stoodley et al., 2010). Also the VI lobule shows some age-related functional changes, since in young adults lobules I–IV, V, and VI are a part of cortico-cerebellar networks involving premotor and primary motor cortical regions, while in older adults the correlations are more widespread, including the hippocampus, middle frontal gyrus, parahippocampal gyrus, anterior cingulate cortex, and dorsal premotor cortex. Therefore, interestingly enough, in older adults the VI lobule is linked to several areas that are crucial for AD cognitive dysfunction.

The selective impairment of Crus I in AD is in line with the role of Crus I as a part of the cognitive “supramodal” zone of the cerebellum, and is in line with previous findings (Guo et al., 2016), though at the present time, this is the first study which assessed the selectivity of the impairment in the progression from a-MCI to AD. The involvement of Crus I is considered to be selective of AD degeneration with respect to different subtypes of FTD (fronto-temporal dementia) (Guo et al., 2016), though a single study reported the involvement of Crus I also in patients with Dementia with Lewy Bodies (DLB) with respect to healthy controls, in contrast with the selective impairment of lobules VI bilaterally in AD patients (Colloby et al., 2014). A recent meta-analysis showed that across different neurodegenerative diseases such as AD, ALS (amyotrophic lateral sclerosis), FTD, PSP (progressive supranuclear palsy), and MSA (multiple system atrophy) atrophy patterns are largely disease-specific, with a major involvement of certain areas of the cerebellar hemispheres such as Crus I/II in AD, ALS, FTD, and PSP and lobules I–IV in MSA and PSP, though with disease specific patterns (Gellersen et al., 2017).

From a functional point of view, there is a clear involvement of Crus I in non-motor functions. Working memory and verb generation tasks activate Crus I bilaterally and mental rotation tasks activate left Crus I (Colloby et al., 2014). Crus I is selectively activated through visuo-spatial cognitive tasks, with a clear lateralization (place-based navigation activates left Crus I, while sequence-based navigation activates right Crus I) with strong cortical projections through hippocampus and bilateral medial parietal and prefrontal cortex (Iglói et al., 2015). Crus I shows highly specific functional connectivity with distributed cerebral regions linked to the default mode network (DMN) (Guo et al., 2016). More in depth, cerebellar regions on the border of Crus I/II are connected to the posterior cingulate, the lateral temporal cortex, the inferior parietal lobule, and an extended region along medial prefrontal cortex (Buckner et al., 2011). Anterior regions of Crus I are functionally correlated to dorsolateral prefrontal cortex, the rostral portion of the inferior parietal lobule, a frontal midline region bordering presupplementary motor area and the anterior cingulate (Buckner et al., 2011). Given the role of the DMN dysfunction in AD and its functional coupling with Crus I, is not surprising that this region shows higher VBM GM atrophy along with clinical progression as showed in our study. Our finding supports the idea that GM atrophy of Crus I is the structural correlate of this functional impairment (Guo et al., 2016), which becomes more evident with increasing disease severity. Moreover we found a voxel-wise association between cerebellar GM changes and a test assessing constructional apraxia in AD dementia patients only.

Constructional apraxia is a neuropsychological landmark of AD, and the correlation between GM atrophy in the cerebellum and lower scores at Drawing Copy test in AD dementia patients supports previous VBM data from our group (Serra et al., 2014). Constructional apraxia could be related to an impairment of basic skills such as visuo-spatial perception and analysis, visuo-motor integration, simple motor skills, or even to impairment in executive functions (such as planning, monitoring responses). The role of the cerebellum in visuomotor integration and in motor feed-forward mechanisms, as well as its involvement in executive functions have been previously demonstrated (Schmahmann and Sherman, 1998), therefore the selective GM atrophy in the “supramodal” lobules (VI lobules and Crus I) of the cerebellum could be reflected from a neuropsychological perspective by an impairment in the Copy of drawings test, as observed in this study. Constructional apraxia is not a cognitive domain that is usually early involved in AD, still it could be the most sensitive in terms of cerebellar contribution to cognition. Though most studies up to now have failed to find an association between MMSE scores and other cognitive or clinical measures in AD and GM cerebellar atrophy (Farrow et al., 2007; Möller et al., 2013; Colloby et al., 2014), our findings might suggest that constructional apraxia might be a useful domain to investigate, although we believe that more specific tests tailored for cerebellar cognitive functions are needed.

## Conclusion

In conclusion, this study indicates a progression of cerebellar GM atrophy from early to late clinical stages of AD, with an early involvement of the vermis and paravermian areas of the anterior (I–V) and posterior (VI) lobes in a-MCI patients, and a later involvement of the hemispheric VI lobules and Crus I in AD dementia patients. Considering the role of the cerebellum in higher-level functions (Strick et al., 2009), we hypothesize its contribution to cognitive decline across AD evolution. Given the selective functional coupling between Crus I and the DMN, and given the selective GM loss in Crus I in AD dementia patients as stated by our work, our results were in line with previous structural and functional findings, though more studies are needed to investigate changes in functional cortico-cerebellar connectivity in the progression from a-MCI to AD dementia.

## Ethics Statement

This study was carried out in accordance with the recommendations of the guidelines for clinical research studies of the IRCCS Santa Lucia Foundation, Rome, Italy, with written informed consent from all subjects. All subjects gave written informed consent in accordance with the Declaration of Helsinki. The protocol was approved by the Ethics committee of IRCCS Santa Lucia Foundation, Rome, Italy.

## Author Contributions

LS and MB conceived the present idea. LS carried out the MRI data acquisition. LS and GO developed the theory and assisted ST in performing data analysis. ST performed the analysis and took the lead in writing the manuscript. MC provided the critical feedback and helped shape the analysis and manuscript. MC and MB supervised the project. All authors discussed the results and contributed to the final manuscript.

## Conflict of Interest Statement

The authors declare that the research was conducted in the absence of any commercial or financial relationships that could be construed as a potential conflict of interest. The reviewer LM declared a past co-authorship with one of the authors, LS to the handling Editor.

## Footnotes

1 http://www.fil.ion.ucl.ac.uk/spm/

## Abbreviations

AD: Alzheimer’s disease
CCAS: cerebellar cognitive affective syndrome
GM: gray matter
HS: healthy subjects
MCI: mild cognitive impairment
MNI: Montreal Neurological Institute
SUIT: spatially unbiased infratentorial template
VBM: voxel-based morphometry
